# From developmental neuroscience to policy: A novel framework based on participatory research

**DOI:** 10.1016/j.dcn.2024.101398

**Published:** 2024-05-31

**Authors:** Yara J. Toenders, Kayla H. Green, Lysanne W. te Brinke, Renske van der Cruijsen, Suzanne van de Groep, Eveline A. Crone

**Affiliations:** a Department of Psychology, Education & Child Studies, Erasmus School of Social and Behavioural Sciences, Erasmus University Rotterdam, the Netherlands; b Developmental Neuroscience in Society, Erasmus School of Social and Behavioural Sciences, Erasmus University Rotterdam, the Netherlands; c Behavioral Science Institute, Radboud University Nijmegen, the Netherlands

**Keywords:** Adolescence, Policy, Youth participation, Societal challenges, Mental health

## Abstract

Insights from developmental neuroscience are not always translated to actionable policy decisions. In this review, we explore the potential of bridging the gap between developmental neuroscience and policy through youth participatory research approaches. As the current generation of adolescents lives in an increasingly complex and rapidly changing society, their lived experiences are crucial for both research and policy. Moreover, their active involvement holds significant promise, given their heightened creativity and need to contribute. We therefore advocate for a transdisciplinary framework that fosters collaboration between developmental scientists, adolescents, and policy makers in addressing complex societal challenges. We highlight the added value of adolescents' lived experiences in relation to two pressing societal issues affecting adolescents’ mental health: performance pressure and social inequality. By integrating firsthand lived experiences with insights from developmental neuroscience, we provide a foundation for progress in informed policy decisions.

## Highlights

• Actively involving adolescents in research could help informing policy.
• Participatory research suits adolescents because of their need to contribute.
• Marginalized communities could be reached through participatory research.
• Policy could be informed through an iterative transdisciplinary research cycle.

## 1 Bridging the gap: a role for adolescents

Adolescent mental health is a well-researched topic in developmental neuroscience. However, the current generation of adolescents is confronted with complex societal challenges, which forces them to navigate in a rapidly changing society (Choudhury et al., 2023). Despite the major research focus on adolescent mental health, less is known about how societal challenges, such as performance pressure and growing social inequalities, affect mental health and associated youth-oriented policies. These societal challenges form a cumulative wave of potential burden that put adolescents at risk, some more than others, for adverse effects on their mental health and developing brain (Poletti et al., 2023, Green et al., 2021, Green et al., 2023a). Therefore, it is of crucial importance that adolescent mental health is studied across societal contexts, to better understand how changes in society affect adolescent development. Subsequently, such scientific knowledge can be a foundation for informing society and policy.

In the present perspectives review, we address a perspective on the role of citizen involvement – particularly the involvement of adolescents - in research and we discuss how citizen involvement may be an adequate mechanism to bridge the gap between developmental neuroscience and policy. Additionally, we discuss why adolescents are particularly suited to inform science and policy, and we discuss how active participation could simultaneously enhance their sense of belonging and well-being. We illustrate this approach within the context of two societal challenges through which the current generation of youth must navigate: performance pressure and social inequality (Choudhury, 2023). Thus, the aim of the current review is three-folded: 1) to highlight *why* adolescents are suited for this role, 2) to illustrate *how* adolescents can actively be involved in research and policy processes, and 3) to reflect on *what* the additional value of youth engaged research could be for developmental neuroscience research, particularly their lived experiences as crucial elements for informing scientific designs and for the translation of neuroscientific findings to policy.

## 2 A neurobiological perspective on why adolescents are uniquely qualified to contribute to science and policy

Adolescence is frequently considered to be a time of vulnerabilities (e.g., onset of psychiatric disorders; Kieling et al., 2024). However, the developmental period of adolescence also poses opportunities (e.g., for contributing to society and prosociality; Crone and Dahl., 2012; Fuligni, 2019). From a neurodevelopmental perspective, the need to contribute to society and to close others might be induced by the puberty-driven development of neural networks and regions that have previously been associated with needs of self and others, such as the ventral striatum (VS), medial prefrontal cortex (mPFC), temporal-parietal junction (TPJ), and dorsolateral prefrontal cortex (dlPFC; Crone and Achterberg, 2022). The VS is a well-studied reward-related subcortical brain region that shows heightened activation in adolescents, compared to children and adults (Braams et al., 2015, Schreuders et al., 2018). Although often linked to negative risk-taking and impulsivity (Casey et al., 2008, Fuhrmann et al., 2015), the VS also has an adaptive function and can promote healthy positive behavior, such as prosociality (Telzer et al., 2013, Telzer, 2016). The mPFC and TPJ are part of the "social brain network", which is a brain network that is consistently involved in new social relations and social decision-making, important processes that take place during adolescence (Blakemore, 2008, Crone and Dahl, 2012). Lastly, the dlPFC is associated with cognitive control processes related to cognitive flexibility and balancing needs for self and others (Do et al., 2019, Crone and Steinbeis, 2017, Crone and Achterberg, 2022). Together, the development of these brain regions forms the neurobiological mechanisms underlying adolescents’ need and capacity to contribute, to act prosocial, and to make impact on the world around them (Fuligni, 2019).

One important characteristic of the adolescent brain is its goal-directed flexibility, particularly as brain regions like the prefrontal cortex (PFC) continue to mature until the early twenties (Crone and Dahl, 2012). As a result, adolescence may be a window of opportunity for social learning, adaptation and creativity, which is defined as the use of original ideas to create new solutions (Nijstad et al., 2010, Kleibeuker et al., 2016, van der Zanden et al., 2020). The PFC is thought to play a key role in creativity and generating innovative ideas, and PFC activity has been found to be enhanced among adolescents when solving problems in experimental tasks with creative solutions (Kleibeuker et al., 2016). Thus, adolescents might be uniquely qualified to come up with innovative ideas to tackle complex societal challenges.

Finally, prior research has emphasized adolescence as a period of exploration and learning (Stevenson et al., 2014). The neurodevelopmental changes related to adolescents’ need to contribute, creativity, and exploration, suggests that adolescents could bring a unique perspective to research and policy (Fuligni and Galván, 2022; te Brinke et al., 2021). Incorporating adolescents’ voices can have beneficial outcomes not only for research and policy, but also for adolescents themselves, as it may fulfill a fundamental need to contribute (Fuligni, 2019).

## 3 How adolescents can be involved: an overview of youth participatory research approaches

Engagement of adolescents in translating research to policy can be achieved via youth participatory research practices (Ozer et al., 2018). Participatory approaches actively involve individuals with lived experiences and insider knowledge (i.e., adolescents, parents, teachers) in research-to-action processes (Vaughn and Jacquez, 2020). In contrast to non-participatory research approaches in which end-users have a passive role (e.g., as subjects in neuroimaging studies or recipients of policy changes), participatory approaches prioritize co-constructing research and policy through active involvement of end-users. The participatory method is grounded in action theories, stating that knowledge constructed without the active participation of affected communities can only be partial (Dedding et al., 2022).

### 3.1 Youth participatory approaches

A range of methods exists for youth to be involved in research (Ozer et al., 2018). According to Hart’s ladder of participation, multiple levels of youth participation can be defined based on differences in the amount of agency, control, and power that adolescents have in a collaboration (Hart and Roger, 1992). At the top of the ladder are youth-initiated activities and shared decision-making, while the bottom of the ladder is comprised of non-participation (e.g., youth are only involved as token, and they have little to no influence). It is not required to always adopt the highest level of youth participation. Here we discuss three examples of youth participatory approaches: (1) youth participatory action research (YPAR), (2) human centered design (HCD), and (3) citizen science. These youth participatory approaches differ in how and to what degree adolescents are actively involved.

**Fig. 1:**
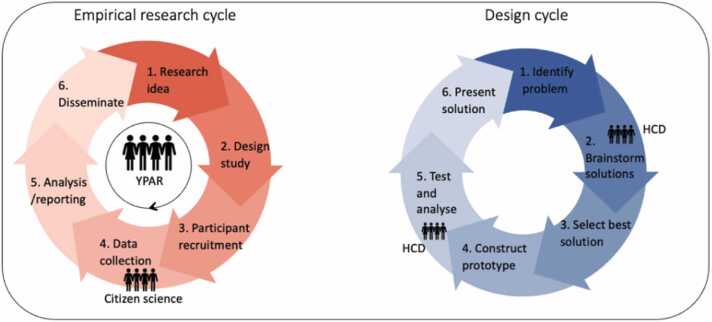
*Youth participation in aspects of empirical and design research.* Empirical (A; left) and design (B; right) research cycles are displayed with the phases during which youth in participatory research can participate. In Youth Participatory Action Research (YPAR) adolescents undergo the entire research cycle, whereas in citizen science adolescents are actively involved only in the data collection phase. In design research, adolescents can participate in the brainstorming and testing phases in Human Centered Design (HCD) research. The size of the figures represents the amount of time and effort the youth put into the participatory research approach.

In YPAR, adolescents are involved as participatory researchers, primarily focusing on topics closely aligned with their personal interests for which they want to contribute to making changes (e.g., climate change; Ozer, 2016). YPAR generally follows the empirical research cycle, encompassing the entire research process from idea generation to research dissemination (Fig. 1A). As such, adolescents are frequently involved in all steps of the empirical research cycle, enabling them to take (partial) ownership of the project, and executing it from beginning to end. HCD has partial overlap with YPAR but has a different starting point. HCD involves end-users in research, as the people who face problems (the end-users) are believed to be the experts when it comes to providing solutions for the problem (Giacomin, 2014). Studies employing HCD follow the design cycle, which involves identifying a problem, developing, and constructing a solution, and presenting this solution (Fig. 1B). Thus, in contrast to YPAR, in HCD studies, adolescents commonly contribute to specific phases of the design cycle, primarily the brainstorming and testing phases. Citizen science is a term often used in adjacent research fields, such as biology (Kullenberg and Kasperowski, 2016), and refers to research where data collection is conducted with participation of non-scientists (e.g., the general public counting butterfly species in their backyard). Citizen science involves, similar to HCD, active involvement in a specific phase of the research. However, as with YPAR, in citizen science, adolescents may take ownership, such as how and when the data collection is conducted. Taken together, these three youth participatory approaches share the commonality of involving youth in scientific research, however, they vary in how and when youth are involved in the process. The additive value of each approach typically depends on the specific research question.

### 3.2 Current examples of youth engagement in developmental psychology and neuroscience

The use of youth participation approaches in developmental neuroscience studies has remained limited, but several studies in other research fields have demonstrated the potential of incorporating YPAR, HCD, and other participatory approaches (Merves et al., 2015), and may therefore also inform developmental neuroscience designs. For example, Kia-Keating et al. (2017) utilized a HCD and YPAR integrative approach to examine health disparities related to violence among Latinx youth. In this study, both youth and adult community members proposed potential solutions, including peer mentorship, parental support, and preventive strategies aimed at fostering a sense of safety and belonging.

In our own studies, we examined how youth can shape scientific questions and designs by actively participating in the research cycle in a multi-sample mixed-method study. In this study, we employed a HCD approach and we collaborated with youth to co-create a novel wellbeing questionnaire (Green et al., 2023b). Through a series of iterative focus groups, youth shared thoughts and perspectives on the development of the Multidimensional Wellbeing in Youth Scale (MWYS). They provided their definition of wellbeing, co-evaluated the questionnaire items and their clarity and relevance, and had the opportunity to add new items. Next, the reliability and validity of the final version of the MWYS was tested. This co-creation process yielded a scientifically robust measure of adolescent and young adult wellbeing that also resonated with youths' own perspectives. This questionnaire is now exploratively used in a fMRI design, making it a co-created fMRI task on wellbeing. A second example from our lab involves the application of citizen science in a study involving optimal social environments for youth. In the ‘All Schools Collected’ study, early adolescents ages 10–13-years were actively involved in the data collection phase as citizen scientists (Toenders et al., submitted). Despite the enormous potential of engaging the wider public in research, citizen science remains uncommon in the social sciences (Heiss and Matthes, 2017), except for the use of snowballing methods, where participants recruit other participants, usually for incentives (Parker et al., 2019). Traditional questionnaire research usually studies social development through the lens of scientists, whereas a citizen science approach, where adolescents collect data, could offer additional insights as it views the social world through adolescents' perspectives. In this project, groups of four adolescents created questions about the social environment of adolescents and subsequently interviewed important adults (key figures) in their lives (e.g., parents, teachers, sports coaches). This not only provided insight into what key figures prioritize in adolescents' social environments but also shed light on what adolescents themselves deemed important. For example, while both key figures and early adolescents rated societal values as important, key figures additionally emphasized the significance of family, whereas adolescents stressed the importance of friends/peers.

Lastly, we incorporated aspects of YPAR and citizen science in a study on positive and negative risk-taking during the COVID-19 pandemic (te Brinke et al., 2022). In this study, youth were actively involved in five phases of the empirical cycle: the research idea, study design, recruitment, data collection, and dissemination phase. Through initial discussions between researchers and actively involved youth, it became apparent that within teen culture, non-adherence to COVID-19 safety regulations was not necessarily viewed as rebellious behavior (negative risk-taking), but also to support each other's need for socialization with friends (positive risk-taking). Thus, active involvement of youth enabled us to examine risk-taking aspects that are defined as socially acceptable or unacceptable by adolescents themselves.

Together, these examples illustrate potential ways of how youth can be actively involved in different phases of the research and design cycle. A next step is to examine how adolescents can bridge the gap between science and policy, through active engagement in the translation of scientific findings into policy.

Box 1 **Considerations for youth engagement in research.**

There are two important considerations for engaging youth in the developmental neuroscience research process. First, when collaborating with adolescents in participatory approaches it is important to take the developmental phase of life they are in into account (Merves et al., 2015). It includes developmental needs and capacities such as agency, independence, short-term vs. long-term thinking, time management, and experiences with vocalizing your thoughts and ideas. For example, whereas research is a relatively slow process (e.g., from an initial research idea to a published manuscript might take years), adolescents’ lives do through may changes already in a time scale of 3–6 moths (e.g., they might not yet know what they will be doing in three months from now). Not taking these time scale differences into account might lead to disappointment and frustration for both sides.

Second, incorporating youth perspectives requires not only action and creativity from adolescents, but also requires societal opportunities, such as accessible education programs, support from family and teachers, and the possibility for adolescents to share their voice in the policy choices (Masten and Motti-Stefanidi, 2020). This approach has benefits not only for youth, but also for the questions or challenges that are being examined, for example, by providing a multi-dimensional perspective on developmental processes to understand the building blocks for successfully growing up, especially given that society is also constantly changing, sometimes rapidly, as in the case of the COVID-19 crisis or the climate crisis. Thus, instead of only focusing on the individual’s capacity for contribution, there is a need to also provide societal opportunities for youth to contribute (Fuligni, 2019).

## 4 Translating developmental neuroscience to policy: a youth participation platform

Overall, there seems to be consensus on the assumption that scientific insights can be meaningful to policy makers. Especially in times of crisis, such as during the COVID-19 pandemic, existing societal problems and their influences on adolescent well-being become more apparent. Yet, the translation from fundamental research findings to operational application in policy can be challenging, because (i) fundamental knowledge about the brain can be abstract and therefore difficult to apply to specific (policy) situations, (ii) neuroscientific findings are often nuanced and prone to misinterpretation (Altikulaç et al., 2019, van Atteveldt et al., 2014, van Atteveldt et al., 2019), and (iii) scientific translation often lacks the lived experiences perspectives of youth and therefore there remains a gap between knowledge and putting knowledge in action. Here, we showcase examples of how youth participation can strengthen the link between scientific knowledge and policy in the context of two pressing societal challenges that affect mental health of youth: performance pressure and social inequality. We address that for the translation to policy, adolescents have a valuable role as their lived experienced with these challenges and their unique perspective on potential solutions may be a missing link. Although involving adolescents may not be the solution to combat all barriers, they can have an important additive value in translating developmental neuroscientific findings to policy.

To inform youth-oriented policy, we developed a framework in which we integrate findings from developmental neuroscience with youth participatory approaches like YPAR, HCD, and citizen science. This framework is embedded in our youth participation platform: YoungXperts. The name of the platform reflects that not we – researchers – but adolescents themselves are the experts when it comes down to their own development. The goals of the YoungXperts platform are 1) to make scientific knowledge accessible to youth and include their perspectives in scientific designs, 2) to collaborate with youth and stakeholders (e.g., youth workers) to integrate this knowledge with their lived experiences and perspectives, and 3) to use developmental (neuro)science to empower the voices of youth and foster positive development. Joining the cycles of empirical research and design provides us with the opportunity for research to increase the potential effect on policy and society. During the different phases of this framework, adolescents are involved through various forms of youth participation. One of the approaches in YoungXperts is to provide youths with ‘FACTS’ based on scientific research (Vallone et al., 2018). The ‘FACTS’ are research findings from recent literature, including both ongoing and finalized findings from international (longitudinal) neuroimaging and behavioral studies. These are subsequently combined with youth’s implementation ideas: ‘TAKE ACTIONS’, which are obtained in focus groups. During the COVID-19 pandemic, this resulted in a youth manifest which prioritized youth’s developmental needs based on scientific findings and adolescents’ perspectives for fitting solutions, which was handed over to the prime-minister and relevant societal parties informing the Dutch cabinet.

In the sections below, we describe how this approach can benefit policy for adolescents’ mental health in the context of two large societal pressures of contemporary times.

### 4.1 Example 1: performance pressure

**Fig. 2:**
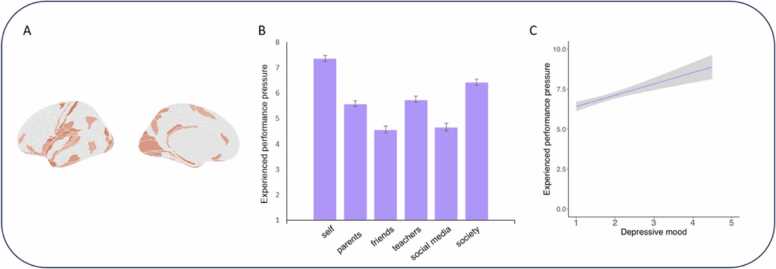
Performance pressure in adolescents. A is an adapted figure from Wong et al., (2023), a study on accelerated brain maturation and stressor reactivity. Stressor load and reactivity was associated with gray matter maturation in regions such as occipital cortex, sensorimotor areas, temporal gyrus, orbitofrontal cortex, DLPFC, mPFC, amygdala, and hypothalamus. B shows the performance pressure youth experience from different targets. C shows the positive association between experienced performance pressure among adolescents and depressive mood state among adolescents. Data in B and C are reported in Toenders et al. (2023).

Performance pressure is defined as the stress experienced to meet society’s expectations (Mitchell et al., 2019; Fig. 2). A threefold increase in performance pressure over the past twenty years has been observed (Curran and Hill, 2022, Stevens et al., 2020). Adolescents may display heightened sensitivity to stressors like performance pressure, due to the impact of these stressors on sleep patterns (Galván, 2020, Yan et al., 2018). Additionally, stress can affect brain development (Sisk and Gee, 2022) and contributes to increased feelings of self-doubt (van der Cruijsen, 2023). There is initial evidence from brain-behavior mechanism insights related to stress that continuous performance pressure can lead to accelerated neural development (Tooley et al., 2021). Because adolescence is an important developmental window for brain development, stress-related alterations in brain development can have adverse effects on their mental health (Uhlhaas et al., 2023). Finally, adolescents are more vulnerable to academic judgement. This is illustrated by self-concept evaluations showing a dip in mid-adolescence when youth are asked to evaluate academic traits (van der Cruijsen, 2018) and brain regions involved in thinking about the self, the medial prefrontal cortex, showing increased activity during adolescence (Pfeifer et al., 2009, van der Cruijsen et al., 2023). These insights from developmental neuroscience indicate that adolescence is a specifically vulnerable period for performance pressure, yet the translation of these scientific findings to policy decisions has remained limited.

Through the YoungXperts platform, we aimed to connect fundamental scientific results on the topics of performance pressure to actionable implementation of ideas from adolescents. In focus group sessions, our aim was to both disseminate research findings and to actively engage youth in addressing the challenges. Depending on the specific aim of the session, different techniques were used, such as design thinking. These co-creation sessions led to the development of concrete action points on various levels, for example for policy, schools, or adolescents themselves. The scientific-facts and take-actions were subsequently shared with youth, policy makers, youth workers, teachers, and scientists. This led to suggested actions by relevant stakeholders, including youth themselves, such as introducing wellbeing classes at schools, reducing the assessment load, and changing the use of everyday language to focus on the learning process instead of performance. These suggestions were combined in a report, together with scientific findings, and presented to the Minister of Education, Culture, and Science of the Netherlands. The report was subsequently cited in a proposed change in policy: to provide students with more time to obtain the number of obligatory study credits that are needed to pass the first year of university in the Netherlands with the aim to reduce performance pressure.

### 4.2 Example 2: social inequality

**Fig. 3:**
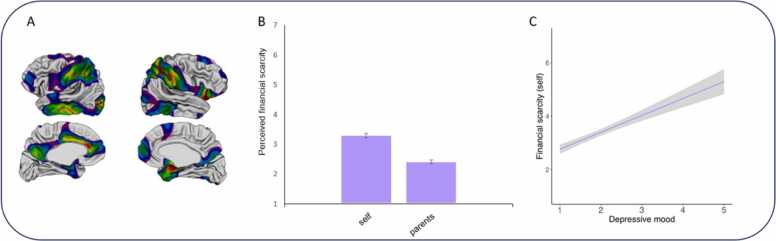
Social inequality in adolescents. A is an adapted figure from McDermott et al., (2019) illustrating the developing cortical surface regions that show a positive association of surface area with SES. B shows the average perceived financial scarcity for the self and among parents as reported by adolescents. C shows the positive association between perceived financial scarcity for the self and depressive mood state among adolescents. Data in B and C will be published in a manifest for policy (Green et al., 2024).

A second urgent societal challenge that today's youth are dealing with, is the increase in socioeconomic disadvantage and social inequality (Patton et al., 2016, Poletti et al., 2023; Fig. 3). Adolescents who are growing up in socioeconomic disadvantaged environments are at risk for mental health issues, both on the short-and long-term (Choudhury et al., 2023, Evans and Kim, 2013, Reiss, 2013, Patel et al., 2018). For example, research on the effects of the COVID-19 pandemic has shown that especially in times of need adolescents living in low-income families – who are facing multiple systemic barriers – are disproportionally hit in their mental health and well-being compared to their more privileged peers (Magson et al., 2020; Green et al., 2023; Stevens et al., 2023). Numerous studies have also shown that experiences of socioeconomic disadvantage and social inequality are associated with alterations in brain structure and function. For example, it has been shown that individuals from lower socioeconomic status (SES) background show accelerated brain development, such that the normative process of cortical thinning is steeper in those with a lower SES (Tooley et al., 2021, Piccolo et al., 2016). Additionally, longitudinal studies have shown disruptions in hippocampal volume among adolescents growing up in poverty (Barch et al., 2020, Noble et al., 2012). On a functional level socioeconomic disadvantage is associated with changes in corticostriatal networks and increased neural activity during mentalizing in the social brain, and to reward in the ventral striatum (Gonzalez et al., 2016, Hyde et al., 2020, Muscatell et al., 2012, White et al., 2022).

Through the YoungXperts platform, we connected fundamental scientific results on social inequality in the school context to actionable implementation ideas of adolescents via focus groups and surveys. These focus groups were organized at schools, and in collaboration with societal partners to reach a diverse group of adolescents. For social inequality, adolescents suggested to increase diversity among teachers, recognize different coping strategies (including accepting social inequality and continue with their lives) or having a personal coach (Green et al., 2024). These take-actions were handed over to approximately 100 teachers in vocational education in an informed debate session and shared through social media channels.

Socioeconomic disadvantage and social inequality are multi-faceted constructs which cannot be captured into single variables, as the aspects vary from racism and discrimination to socioeconomic status, stress about finances, growing up in poverty, and residing in less resourceful neighborhoods (Sheridan, 2023). To address these challenges, it is essential that (neuroscientific) research explicitly takes systematic barriers into account. This involves adequately empowering adolescents to express their perspectives, as they grow up during these societal challenges which potentially affect their mental health. Therefore, policy implications of developmental research should be considered together with youth who are affected by the outcomes of the research (Dahl et al., 2018).

## 5 The beneficial effects of youth participatory approaches

Engagement of adolescents in translating research to practice and vice versa, and inclusion of youth’ lived experiences seems a promising avenue to facilitate the uptake of neuroscientific findings in policy (Fuligni and Galván, 2022). Prior research has explored the potential beneficial effects of youth participatory research and demonstrated benefits on multiple levels. Although most developmental studies in which youth participatory approaches are used are primarily focused on behavioral processes, the benefits discussed below are also applicable to developmental neuroscience.

### 5.1 Benefits for researchers

Participatory youth panels may provide a good solution to integrate empirical evidence with the interpretation of youth on existing problems (Jacquez et al., 2013, Ozer et al., 2020). First, participatory research allows researchers to reach marginalized communities that are not typically represented in research (Vaughn et al., 2013). Scientific results are often based on a biased sample, for example, youth with good reading and attention skills (Fakkel et al., 2020). These biased samples may not reflect the perspectives of the entire population of youth. Reaching and including previously excluded adolescents in research may be beneficial for generalizability of findings, as well as validity, reliability, and reproducibility of measurement instruments and results, and should therefore be an important aim for researchers (Garcini et al., 2022, Green et al., 2022). Participatory approaches facilitate reaching and empowering a diverse group of adolescents (Maker Castro et al., 2022). Succeeding in these efforts requires building trust in communities in which equal relationships are developed between researchers and stakeholders (e.g., youth). Next, youth engagement can reshape the traditional power dynamics between researchers and participants. This shift could be particularly valuable for adolescents who are traditionally underrepresented in research (Postma, 2008). Third, research into society-oriented actions is influenced by personal and generational goals, therefore, lived experience is crucial. Finally, active involvement can increase citizenship skills and may lead to translation of the research to a change in policy (Jacquez et al., 2013, Zeldin et al., 2007). Suggestions for how youth participation can be applied in developmental neuroscience studies can be found in Box 2.

Box 2 **Suggestions for youth participatory approaches in developmental neuroscience.**

– Collaborate with adolescents to develop and improve relevant research questions that might be answered using neuroscience
– Co-design experimental tasks for in the fMRI scanner together with adolescents
– Discuss together with adolescents about how to improve MRI sessions to increase reliability, and how to reach a diverse group of young people to come to the scanning facility to increase generalizability
– Work with adolescents (youth ambassadors) to reach new participants. Many adolescents are already trusted by their peers/community and might therefore be able to reach a more diverse group of adolescents
– Organize focus groups on how to interpret findings, what does this mean in the context of today’s society? How do adolescents with lived experience look at these findings?

### 5.2 Benefits for policy makers

Second, it is argued that participatory approaches can lead to increased societal impact, including youth-benefitting policy (Kirby and Bryson, 2002). In a systematic review on the outcome of youth inquiry approaches such as YPAR, youth organizing, and youth advisory boards, researchers identified 61 youth inquiry studies in the United States (Kennedy et al., 2019), focusing on topics like bullying prevention and substance use. Of these 61 studies, 5 led to policy development (8.2 %) and 19 led to formalized program development or enhancement (31.1 %). Policy changes will have more impact when they are based on participatory scientific research and when there is already support from stakeholders. Policy makers will also benefit from providing input at the initial phases of the scientific process to inform scientists and stakeholders about potential restrictions as well as opportunities for implementation.

### 5.3 Benefits for youth

Youth participatory approaches have the potential to benefit developmental neuroscience and policy, but also adolescents themselves. Lastly, a potential benefit of participatory approaches is that active involvement allows for research methods that are better tailored to the needs of youth, aligning with their developmental requirements (Abraczinskas et al., 2022). Second, active involvement of adolescents in research can empower youth and enhance their self-esteem (Head, 2011, Sabo, 2001), particularly in settings where their agency is constrained by hierarchical structures, such as schools. YPAR has previously been shown to empower youth by positively affecting youths’ motivation to influence their school or community, their participatory behavior, and their socio-political skills.

## 6 Future directions: transdisciplinary approaches and Living Labs

**Fig. 4:**
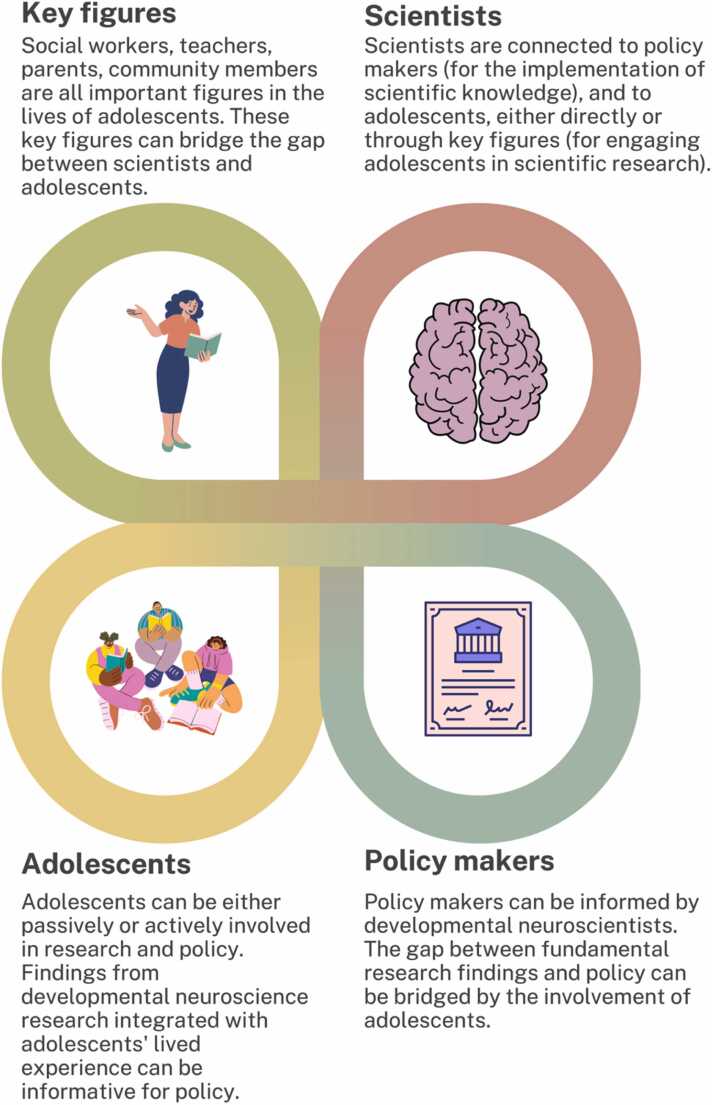
Conceptual model of an iterative process between researchers, key figures, youth, and policy makers.

Youth participation approaches, like the YoungXperts platform, may provide new societal opportunities for adolescents’ contributions, potentially leading to better implementation of science into policy. This approach fits with a larger trend to extend our research to include not only interdisciplinary, but also transdisciplinary approaches. Transdisciplinary science is defined as the cooperation among different scientific domains to solve highly complex interconnected societal challenges, in collaboration with societal partners, to foster impact-driven changes (Brandt et al., 2013). It not only crosses scientific disciplines (i.e., interdisciplinary science) but also uses knowledge coming from societal contexts, such as citizens (i.e., youth as experts) and/or societal organizations (i.e., mental health care organizations, community centers). A step further in the transdisciplinary framework is to involve not only adolescents in research, but also other societal stakeholders, including youth organizations, health organizations, and policy makers from the start to in order to conduct neuroscientific research in line with societal needs (Fig. 4). Such alignment of research objectives can serve as the foundation for translating collaborative research findings into policy. As the problem is formulated collaboratively at the start, transdisciplinary approaches lead to ‘co-ownership’ of the problem by multiple stakeholders, which facilitates subsequent ownership of the results and the need for action (Fig. 1). Especially in times of societal challenges, it becomes more pressing to not only produce scientific evidence, but also to put scientific evidence into action (Clark et al., 2020). A transdisciplinary developmental neuroscience approach can provide unique insights into adolescents’ needs and give adolescents opportunities to develop into engaged, contributing members of society. Thus, there is a sense of urgency that science and policy can inform each other for the benefit of adolescent health (Suleiman and Dahl, 2017).

One potential step in this direction is through the implementation of Living City Labs, defined as geographically bound workspaces (such as a city or a neighborhood) where multiple parties collaborate with their own roles and responsibilities. In a Living Lab approach, scientific and societal problems are approached using an iterative design-thinking approach, which allows for adjustments based on user feedback. Living Labs have been widely used for science-business collaborations in life science and technology, but recently there is a growing trend towards their application in the field of social sciences (Dekker et al., 2020). An advantage of a Living City Lab is that these labs facilitate collaboration among scientists, citizen organizations, societal partners, adolescents, and policy. When there is a foundation of mutual trust and clear delineation of responsibilities, transdisciplinary science can promote opportunities for better mental health care for youth (such as within school programs) and in addition provide avenues for adolescents to be heard and contribute effectively (te Brinke et al., 2022).

## 7 Conclusion

This review aimed to provide a novel perspective on policy implications based on developmental neuroscientific findings in the context of the current societal challenges. In recent years, numbers of mental health problems among youth are rising and researchers have argued that the current societal challenges, such as increasing performance pressure and growing social inequality, reinforce the need to provide societal opportunities that increase adolescents’ vigor, sense of belonging, and wellbeing (Fuligni and Galván, 2022).

We argued that adolescence may be a sensitive window in the development of action-oriented behavior and creativity that affects not only adolescents individually, but also has the potential for youth to be agents of societal change. We reviewed evidence from experimental-, neuroimaging-, survey-, and youth-panel research demonstrating that combining these approaches enables researchers to answer complicated questions regarding adolescent development. An important message for scientists is therefore to enrich our concept of adolescent development, with the lived experienced and perspective of youth. This will allow us to test our theoretical models accordingly, and adapt theories if necessary.

The current global challenges underline our need for new ideas on how youth can actively contribute to pressing societal challenges (Wray-Lake et al., 2016). We argue in this review for a comprehensive transdisciplinary approach using complementary methods, and for a richer assessment of the potential of adolescents to contribute to these challenges. In this way, the translation of neuroscientific findings can be combined with the lived experiences of adolescents, resulting in valuable insight for policy implications.

## Funding

This study was funded by the NWO Spinoza prize awarded to Eveline A. Crone, the European Research Council (ERC) under the European Union's Horizon 2020 research and innovation program (grant agreement nr. 681632), Muller Foundation, a named fund at the 10.13039/501100005797Erasmus Trustfonds, and the Nationale Wetenschapsagenda.

## CRediT authorship contribution statement

**Yara J Toenders:** Conceptualization, Visualization, Writing – original draft. **Suzanne van de Groep:** Writing – review & editing. **Renske van der Cruijsen:** Writing – review & editing. **Lysanne W. te Brinke:** Conceptualization, Writing – review & editing. **Kayla H. Green:** Conceptualization, Visualization, Writing – original draft. **Eveline A. Crone:** Conceptualization, Funding acquisition, Writing – original draft.

## Declaration of Competing Interest

The authors declare that they have no known competing financial interests or personal relationships that could have appeared to influence the work reported in this paper.

## Data Availability

No data was used for the research described in the article.
